# The most sensitive inputs to cutaneous representing regions of primary somatosensory cortex do not change with behavioral training

**DOI:** 10.14814/phy2.12623

**Published:** 2015-12-03

**Authors:** David T. Blake, Elsie Spingath

**Affiliations:** ^1^ Department of NeurologyBrain and Behavior Discovery InstituteAugustaGeorgia

**Keywords:** Implant, map plasticity, receptive field, somatosensory

## Abstract

Learning a sensory detection task leads to an increased primary sensory cortex response to the detected stimulus, while learning a sensory discrimination task additionally leads to a decreased sensory cortex response to the distractor stimulus. Neural responses are scaled up, and down, in strength, along with concomitant changes in receptive field size. The present work considers neural response properties that are invariant to learning. Data are drawn from two animals that were trained to detect and discriminate spatially separate taps delivered to positions on the skin of their fingers. Each animal was implanted with electrodes positioned in area 3b, and responses were derived on a near daily basis over 84 days in animal 1 and 202 days in animal 2. Responses to taps delivered in the receptive field were quantitatively measured each day, and receptive fields were audiomanually mapped each day. In the subset of responses that had light cutaneous receptive fields, a preponderance of the days, the most sensitive region of the field was invariant to training. This skin region was present in the receptive field on all, or nearly all, occasions in which the receptive field was mapped, and this region constituted roughly half of the most sensitive region. These results suggest that maintaining the most sensitive inputs as dominant in cortical receptive fields provide a measure of stability that may be transformationally useful for minimizing reconstruction errors and perceptual constancy.

## Introduction

Sensory signals in touch initiate at the finger tips, proceed through two synaptic stations, before arriving at the cerebral cortex. There the incoming signals terminate in the middle cortical layers and activate entire columns of neurons (Mountcastle 1957). The fine organization of the hand map includes adjacent and largely non–overlapping suprathreshold, or action potential‐based, responses for each digit (Merzenich et al. 1978). Interleaved in the hand map are patches of representation of the hairy skin. The system is exposed to challenges throughout life and adapts to these challenges. In response to a loss of inputs, that is, in digit amputation, the deafferented cortical region becomes activated by immediately adjacent inputs (Merzenich et al. 1984). If digits are altered to prevent independent movement in syndactyly, the representational borders between the digital representations are erased (Allard et al. 1991). Operant training induces behaviorally relevant skin surfaces to expand in their cortical representations (Jenkins et al. 1990; Recanzone et al. 1992b; Xerri et al. 1999). If an animal responds to stimuli that activate the adjacent digits simultaneously, the borders between digital representations are similarly erased (Wang et al. 1995b). These observations led to a theory that Hebbian principles provided a first approximation to understanding the principles governing cortical reorganization in the adult (Buonomano and Merzenich 1998). However, further studies have demonstrated conclusively that Hebbian principles, if they are active at all, are subjugated to other dominant reorganizational principles (Blake et al. 2002b, 2005, 2006; Carpenter‐Hyland et al. 2010; Spingath et al. 2011, 2013).

First, a search for a governing time window for Hebb‐like processes underlying reorganization found that the time constant of integration for such reorganization had to be at least 100 msec, which exceeds the timing at which adjacent digits are typically activated in processes like typing (Blake et al. 2005). Second, learning to detect a new target stimulus, under a Hebbian prediction with reinforcement, would selectively increase the responses of the coactivated neurons, or those that represented the target. Instead, learning to detect a new target causes response enhancement that is nonselective. In any Hebbian model, the neural activity must be central to the plasticity. Our new theory holds that learning triggers an increase in cortical responsiveness that is responsible for changes in cortical representations (Spingath et al. 2011, 2013). This prior work has established that in a 2‐week period after learning a sensory detection task, cortical responses and receptive field sizes nonselectively increase in response strength. In the 2‐week period after adding a task distractor, the neural responses to the task distractor decreased selectively. The neural responses during behavioral performance were 30% stronger than the neural responses during passive restraint prior to the behavioral session. During behavioral performance, the neural responses to the task distractor showed signs of longer latency suppression which may guide the selective suppression of the responses to the distractors. These findings, from prior work that was based on these same experiments (Spingath et al. 2011, 2013), are extended in the current study.

A major remaining question is how the fine structure of the cortical map may be altered by this learning. Do the representations of the task distractors and targets warp the cortical map by shrinking and enlarging those representations (Jenkins et al. 1990; Recanzone et al. 1992b; Xerri et al. 1999, 2005)? Or does plasticity primarily show in changed responsiveness without changes in map structures? In the current study, neural responses from electrodes implanted into primary somatosensory cortex were tracked throughout the duration of study, over 200 days in one animal. These responses included at least one round of detection/discrimination learning, and in some cases three. Each round of learning was at least 6 weeks, or 30 recording days, long. We show that the most sensitive zones in the receptive field at each cortical location have subregions that are present on all days in which receptive fields are measured. Flanking those subregions are other portions of the sensitive regions that are variable from day to day. The variable subregions change slowly, with some preservation of the variable subregion for at least 3–4 months.

## Materials and Methods

### Physiological recordings

Full methods are detailed in a prior publication (Spingath et al. 2011). Portions of those methods relevant for the scientific questions in this work are included here. All data in this work were obtained from two adult, male Rhesus macaques weighing 4–7 kg. They were each implanted with an array of 64 microelectrodes. The microelectrodes were implanted into the somatosensory cortex. The somatosensory cortex was localized physiologically with microelectrode penetrations in surgery under barbiturate anesthesia to localize cutaneous somatosensory digit responses in the central sulcus, with the search for responses initiated at +6 mm anterior and 24 mm lateral. Electrodes were implanted into area 3b. Microelectrodes were parylene‐insulated iridium or parylene‐insulated platinum–iridium electrodes that tapered from a 40 *μ*m diameter to an exposed electrode tip that ranged from 5 to 7 *μ*m long. This length of tip exposure was used to allow sampling from the smaller cell bodies present in sensory cortex (Hubel 1957). Our implants are custom made to allow sampling from independently vertically positioned electrodes designed for implantation (Schmidt et al. 1988). Electrode depths were optimized for recording in the 6‐week period after implantation surgery. After that point in time, electrodes were left unmoved for the remainder of the data presented in this work. Cortical implants are adapted from methods described previously (de Charms et al. 1999). Significant alterations to this method consisted of adding a fluid drain to relieve potential hydrocephalus (Miyakawa et al. 2012), removal of the dura in surgery in the areas of future electrode penetration, and the replacement of cyanoacrylic bone cements with INFUSE bone graft (Medtronic) and autogenous bone chips. Spike detection thresholds were set manually on each channel so that spontaneous rates were roughly 10–20 Hz or roughly 3.75 standard deviations of the noise.

Receptive fields were defined using hand‐held 1‐mm rounded glass tipped probes. Skin areas were included in cutaneous receptive fields if just‐visible indentations of the skin evoked consistent audible responses in 250–10,000 Hz filtered voltage signals from the electrode. Calibration of this method with displacement controlled stimuli has determined that this threshold is under 100 *μ*m. Stronger stimuli were used to map deeper or weaker contributions to the receptive fields which were separately noted. Pacinian input was determined by poorly localized, highly sensitive inputs to the glabrous skin, and hairy skin inputs were determined by responses to movements of isolated hairs. Receptive fields for analysis did not have Pacinian, proprioceptive, or hairy skin responses. Trapezoidal skin indentations were not used to separate RA and SA1 inputs, and recent evidence casts doubt on separate processing channels for SA1 and RA inputs in primary somatosensory cortex (Pei et al. 2009). Using Reconstruct software (Synapse Web, Austin, TX), receptive field boundaries were drawn over images of the hand and digits, and receptive field sizes were calculated by the software. Collection of automated receptive fields is not trivial in the somatosensory system, although it has been performed over a limited glabrous skin surface for peripheral afferents (Johansson 1978), and over planar surfaces in central neurons (Killebrew et al. 2007). Receptive field maps over highly curved portions of the finger may be derived easily manually, but are especially challenging to do in an automated setup. Cutaneous receptive fields in this study were contour mapped using five grades of sensitivity in their driven response. At Grade 5 the oscilloscope typically showed one or more cleanly isolatable single units for study, and just visible indentations of the skin with a glass probe elicited vigorous responses. The Grade 4 or “clearly cutaneous” sites were vigorous, but below the level at which Grade 5 or “very responsive” sites were judged. The Grade 3 or “just cutaneous” meant that consistent responses to just visible stimulation of the skin were elicited, but that if responses were any weaker, then they could not be judged to be consistent. Grade 3 implies a reasonable confidence that the responses were based on input from low‐threshold SA1 or RA peripheral mechanoreceptors. Grade 2 responses were consistent, but not responsive enough that it can confidently be determined that low‐threshold mechanoreceptor input is required for the response. Grade 1 is even more sluggish than Grade 2, but localized enough that an assessment may be recorded. For comparisons in which the most sensitive portion of the receptive field were compared, only the highest grade contour present for that site were used each day.

The person mapping receptive fields were the same throughout all studies, and were blinded to the identity of the electrode being mapped.

### Stimulus presentation

**Figure 1:**
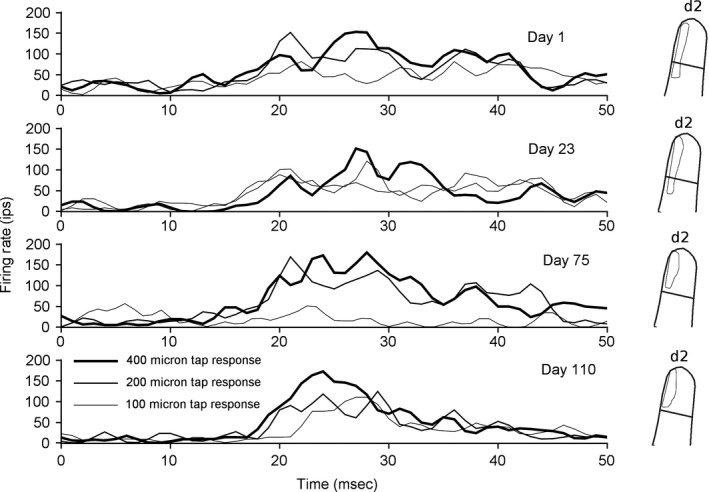
An example site. Each graph shows the averaged neural responses from one cortical location in one day's recordings. Responses to three different amplitude taps are overlaid, and are shown for each of four different days. On the right, the contour of the most sensitive region of the receptive field on that day is shown.

Somatosensory stimuli were delivered via custom‐built tactile motors under LVDT displacement‐feedback control. These motors were built to mimic the Chubbuck motors, except that they are smaller and deliver displacements with a 0.5‐mm range (Chubbuck 1966). Each tap delivered from the motors was a single period of a 40‐Hz raised sinusoid with a phase of −p/2 at its start and lasting 25 msec. More simply, a smooth tap with zero first derivative at the start, end, and midpoint. Neural responses to motorized taps were recorded before the day's behavior, during the behavior, and after the behavior in awake Rhesus macaques. This work only presents data in response to the motors in Figure 1. The motorized tip was lowered until barely touching the skin and then indented 500 *μ*m into the skin before delivery of any taps.

### Animal behavior

Animals were trained in three tasks serially for weeks: lever holding, detection, and discrimination. A complete experimental run consisted of an animal performing one run of lever holding, detection, and discrimination, and it required 1–2 months to complete. Time to completion depended on how long the animal took to learn the tasks. Each task was performed for at least 2 weeks before the animal was moved on to the next behavioral task.

The present dataset includes samples taken before any task learning, before detection learning, after detection learning, after discrimination learning, and in between periods when the target was moved from one skin location to another. Behavioral effects on neural responses are detailed in two prior works (Spingath et al. 2011, 2013). The present work focuses on aspects of the neural response that do not change with learning, and these aspects of the responses have not been considered in detail in prior work.

Animal welfare was regulated by the Institutional Animal Care and Use Committee at the Medical College of Georgia.

## Results

The purpose of the present work was to reconstruct the responses at single cortical locations over the entire length of study, and to look for patterns of stability and plasticity in neural response strength and receptive field structure. In the two animals, recordings and receptive field maps were derived from 63 implanted electrodes. Nineteen of these had consistent cutaneous responses throughout recording periods, and over 500 receptive field profiles and quantitative firing rate profiles were taken. One example, shown in Figure 1, shows responses taken from one electrode, and peristimulus time histograms from stimulation of one location on the skin at four time points separated by more than 100 days. The basic finding of this work is that the most sensitive portion of the receptive field contains a subregion that is present on all days. This result occurs throughout periods of detection and discrimination learning that have described impacts on neural receptive field size and sensitivity (Spingath et al. 2011).

The methods used for receptive field estimation are manual. Each day, an investigator (E.S.) listened to the activity from each electrode filtered between 250 and 10,000 Hz. The investigator was blinded to the identity of each electrode and had to search the entire hand. Using glass‐tipped probes, the receptive field was examined and overlaid onto a computer image of the hand. Cutaneous ratings, or sensitivities 3, 4, or 5, were used when the skin yielded a consistent response to just visible skin indentations. The sensitivity of 3 is the lowest sensitivity at which investigators are confident that input must come from peripheral SA1 or RA cutaneous mechanoreceptors.

The animals were pretrained to allow investigators to manipulate the digits without resistance. This training consisted of rewarding the animal with food while its hand was gently manipulated. Over some time, the animal allows the gentle digit manipulations necessary to map receptive fields without resistance. In most cases, receptive fields were easily mapped without digit restraint. The investigator also had to map hairy receptive fields, and proprioceptive inputs, which are difficult to evaluate if the digits are restrained.

This method has been calibrated using custom piezoelectric stimulators (D.B.) to mean that cutaneous ratings are delivered when a brief 40 *μ*m stimulus elicits consistent, audible responses. Responses that were manually rated as weaker than cutaneous rarely had structured responses to taps of 400 *μ*m in their receptive field, whereas those rated cutaneous almost always did. The investigator typically rated 20 electrodes in a 30‐min period. The same electrode was occasionally tested more than once to evaluate reliability.

**Figure 2:**
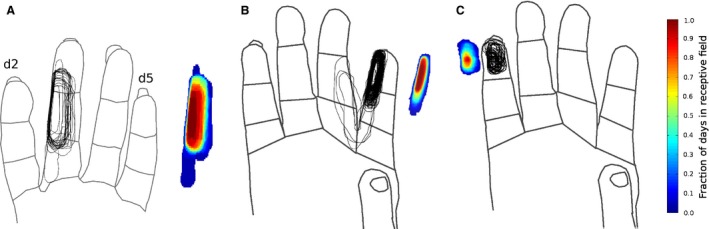
(A) Receptive fields from one electrode. Contour lines over the hand trace the most sensitive skin inputs to one position in primary somatosensory cortex, 25 samples over 31 days. The heatmap net to each hand shows the probability of i different locations being present on any 1 day. Samples were from animal 1. (B) A similar set of plots from one location in animal 2, with 121 receptive fields over 202 days. (C) A third example, from animal 2, with 43 receptive fields over 146 days.

Receptive fields from the same implanted electrodes were measured every day for the duration of study, shown in Figure 2. The figure overlays the highest rated portion, or most sensitive zone, of the receptive field on each day. To the right of the receptive fields is a color map indicating the percent of the days that each position on the skin was present on the receptive field. In examples in Figure 2A and B, a significant portion of the receptive field area is conserved over time. This specific finding was also true in three of the other four cases in which more than 25 receptive fields were associated with an electrode over the length of study, and the sensitivity of those receptive fields was 3 (cutaneous) or higher on each day. The one example in which this did not hold true is shown in Figure 2C. All the receptive fields occurred on the same digit segment. Although there was not a skin region present on each day, the same skin segment was found in over 90% of the sample. A later section considers responses evaluated at less sensitive than a cutaneous rating. The null hypotheses that these receptive fields are randomly drawn can be rejected. Also, these results require testing two other pairs of hypotheses. Is the cortical map unstable or stable? Are the electrodes moving and sampling different neurons over time, or are they sampling the same neurons? For the former, we draw upon the cortical columnar rule (Mountcastle 1957; Powell and Mountcastle 1959). If we sample neurons separated by more than 600 *μ*m in a direction parallel to the cortical surface, their receptive fields will not overlap. Our electrodes are oriented parallel to the cortical surface, and receptive field overlap is found throughout study. Accordingly, the map's representations must move less than hundreds of microns throughout study.

**Figure 3:**
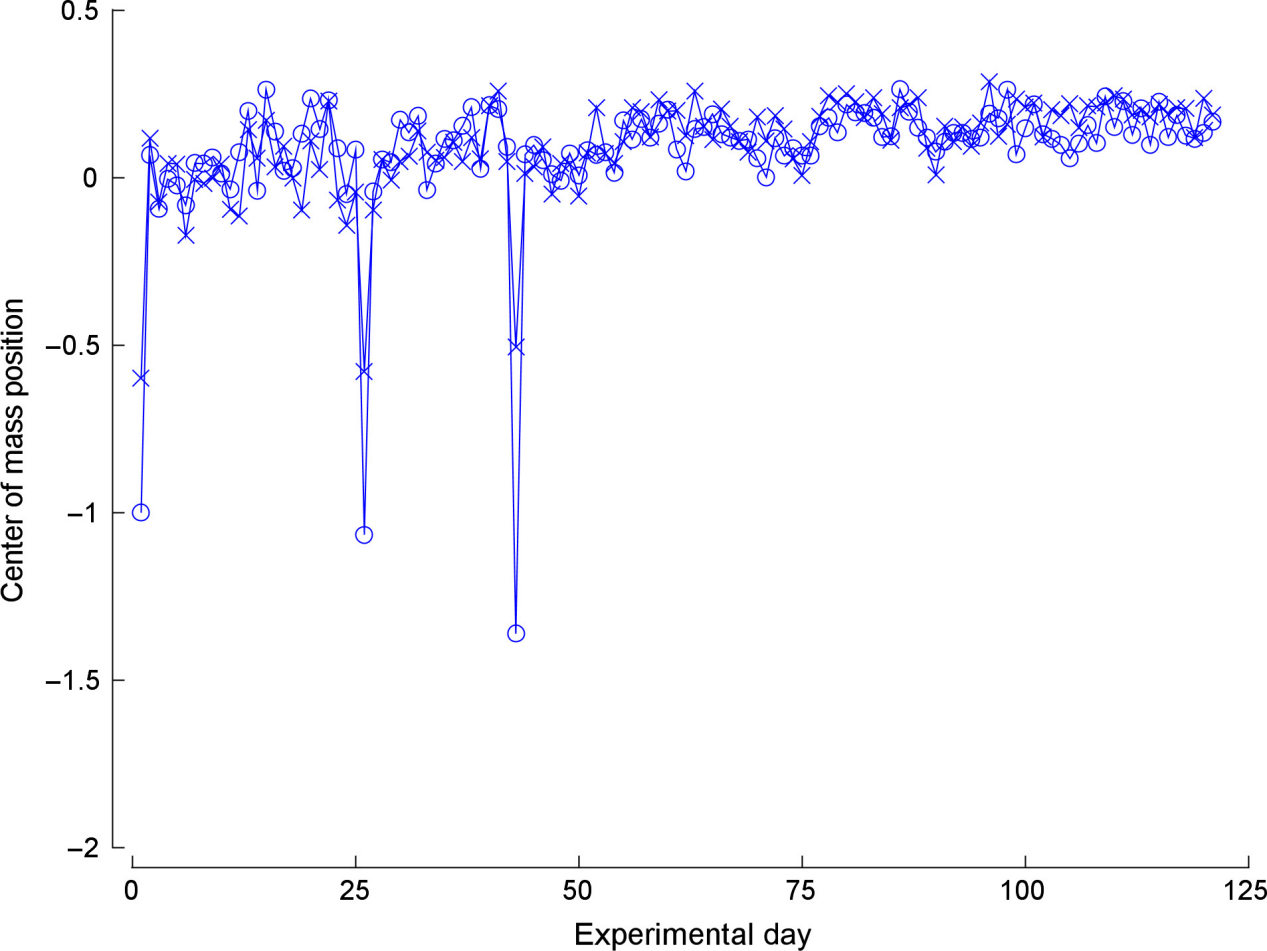
Center of mass movement. The lines joined by circles plot the movement of the *x* coordinate of the receptive field center of mass, while the lines joined by *x* plot the *y* coordinate. Each position is normalized with zero as the initial position, and 1 means the center of mass moved the mean receptive field range in that coordinate.

To more carefully consider the hypothesis that the electrodes may be moving, we calculated the center of mass of the most sensitive receptive field region for Figure 2B and plotted its X and Y coordinate over time. The coordinate system was normalized by the mean range of the receptive field on each axis in order to make movement of magnitude 1 equal to the distance of one receptive field. As shown in Figure 3, the mean distance moved is approximately 17% of one receptive field in each coordinate, or roughly 25% of one receptive field diameter if both coordinates are considered. On the basis of the cortical columnar rule, the movement of the center of mass is much less than 600 *μ*m. Similar calculations were performed for the other five electrodes with consistent cutaneous responses with similar results.

**Figure 4:**
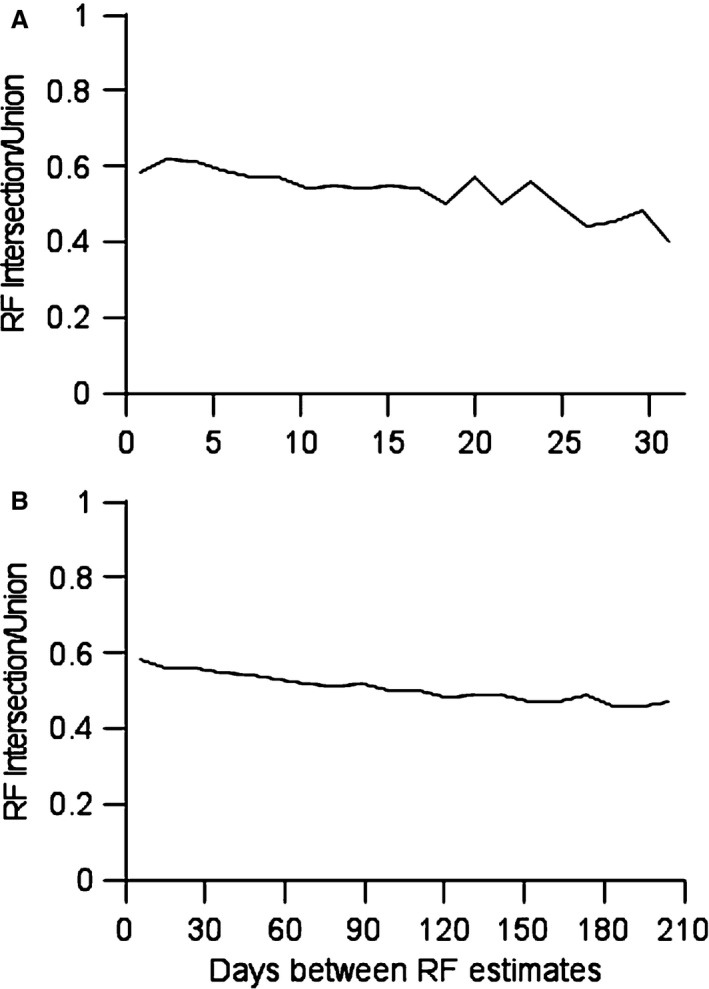
(A) Ratio of receptive fields’ intersection to union. Pairwise samples from single locations in animal 1 were used to calculate receptive field intersection to union ratios. Values averaged above 0.5 if pairs were sampled 1 day apart, and decreased for longer time differences. (B) Data from animal 2.

A measure of receptive field overlap can also aid in estimating the distance moved by an electrode. The average receptive field overlap from samples taken from the same electrode is plotted in Figure 4. Plotted as a function of time between samples, the measures from each animal are above 0.5 for short time separations, and closer to 0.4 for long time separations. The presence of an absolute stable component in the most sensitive region in the receptive field would mean the intersection to union ratio would asymptote at a positive value. In contrast, the ratio would asymptote to zero if no stable region exists. The samples were grouped into 20 time groups per animal for statistical purposes, and the changes as a function of time were significant in each animal (Kruskal–Wallis test, *n* = 638 pairwise combinations, *P* < 0.006 in A or animal 1, and *n* = 8400, *P* < 10 14 in B or animal 2). The ratio decreases as a function of time, but is still significantly larger than zero. These observations are consistent both with more receptive field similarity at shorter intervals between observations, and with the presence of a stable invariant component to the most sensitive region of the receptive field.

The data from Figure 4 can be used along with the published data to estimate the possible distance the electrode tips may have moved over the course of the experiment under the assumption that the responses were absolutely unchanging over time. Average receptive field overlap at a distance of 200 *μ*m is 0.2 (Blake et al. 2002a). The present work finds receptive field overlap at the longest time points assessed is 0.4. Given the assumed concavity of the overlap function, this would correspond to a distance close to 100 *μ*m. This distance estimate can be used to estimate the sampled volume used to generate the most sensitive inputs at each site. We assume each electrode can “listen” to electrical currents in the neuropil for a fixed spherical distance. If the electrode were to move over time, the sampling volume would be a cylinder with a hemisphere on each end. The cylinder would have a radius of approximately 20 *μ*m, a length of 100 *μ*m, and a hemisphere radius of 20 *μ*m on each end. The estimated sampled volume would be 0.000161 mm^3^. Using the estimated neuronal densities of 80,000/mm^3^ (Collins et al. 2010), the electrode had an opportunity to sample from about 13 neurons if it moved 100 *μ*m in a straight line. The assumption of a sampling distance of 20 *μ*m is based on the size of neuronal soma in area 3b, which is generally under 20 *μ*m, and the biophysical estimate that cells that are predominantly stellate, as they are in area 3b, may be sampled above the noise only when the electrode tip is within roughly the distance equal to one diameter of the cell body (Boulton et al. 1990). An analogous comparison would be that we are assuming a single neuron in area 3b can be sampled effectively for at most 40 *μ*m of one electrode's track. It is reasonable to conclude that each electrode is sampling in its most sensitive region 1–4 neurons on any given day, with a total sample number of 10–20 neurons sampled from a small volume over the length of study.

An assumption in calculating the number of sampled neurons is that the structure of the inputs does not change. If neuroplasticity caused changes in the spatial structure of the most sensitive inputs, these numbers would be even lower. For example, if neuroplasticity caused one half of the receptive field to disappear, the overlap ratio would decrease to 0.5, and the center of mass would move 25% of the receptive field diameter, and this would occur only if the same location were sampled (the same 1–4 neurons throughout study). Any variability in the spatial structure of the inputs over time would cause receptive field overlap to be lower, not higher, which would indicate the number of neurons sampled would be smaller. Similarly, the manual mapping methods may be challenged as inadequately quantitative, but any variability introduced by this methodology would cause receptive field overlap to be lower, not higher, and thus strengthens the argument that the electrode movement over study is minimal and a portion of the inputs stable. The finding, then, is that a portion of the most sensitive inputs in a small volume sampled by one electrode is not changing over time. This finding is restricted to those cortical locations in which electrodes sampled from neurons with cutaneous response properties more than 25 times during study.

To extend these findings to the other locations at which data were collected, another 13 locations were added to the analysis, five from animal 1 and eight from animal 2. Each location had at least 10 days with receptive field sensitivity of 2 or greater. Two locations failed to meet the criteria of always containing a constant subregion in the receptive field. The other 11 locations had regions that were always present. On the two locations that failed to have an invariant region in the receptive field, the mapped sensitive region was largely confined to a single digit, although it was proximal on some days, and distal on other. These receptive fields, with a maximal sensitivity rating of 2, do not respond consistently to just visible indentations of the skin at any location, and thus require more vigorous stimuli to map. The use of more vigorous stimuli to map both increases variability and decreases the confidence of the receptive field mapper in the selectivity of the stimulation.

Sixty‐four electrodes were implanted in each animal, yet data supporting consistency in the spatial location of the most sensitive inputs is only provided on 19 electrodes. What about the other 109 electrodes? In our implants, roughly half of the electrodes do not ever yield recordable signals. In this case, 63 electrodes had driven responses at some point in training. Because of the geometry of area 3b, recording electrodes are parallel to the cortical surface. Electrodes in the upper or lower layers of cortex yield much less consistent recordings than those in the middle lamina, and thus rarely sample frequently enough to be included in this analysis. Some electrodes, in addition, only yielded deep responses, or proprioceptive responses, or had receptive fields that were not easily used in our study (like on the wrist or hairy skin). No observations occurred that would run contrary to the hypothesis that a subset of the most sensitive inputs at almost every cortical location with cutaneous inputs is impervious to behavioral training. Although we can only quantify observations at locations with suitable recording quality, we failed to observe any real shifting of the most sensitive inputs that may underlie instability in the cortical map.

## Discussion

The current work augments our knowledge of the principles governing neuroplasticity induced by learning. Specifically, responses at single locations in sensory cortex may be altered in responsiveness and receptive field size (Spingath et al. 2011), while changes in the most sensitive inputs are more restricted. Our observations are restricted to the task we chose to use, although other tasks have yielded similar, but less thorough, results (Blake et al. 2005). All the cortical locations that were consistent in responsiveness and sensitivity maintained a fixed locus of peripheral epithelia as a portion of the inputs to their receptive fields. From the analysis on the ratio of receptive field intersection to union, the magnitude of this subset may be estimated. On average, a ratio of 0.4 between receptive field intersection and union would correspond to the stable locus of inputs constituting 57% of the most sensitive portion of each receptive field.

An issue in the interpretation of these results is that of sampling bias. Locations were selected based on consistency and sensitivity, so these results hold for the most active and responsive locations in sensory cortex. It is certainly the case that many sites responded less vigorously and fell below the threshold for inclusion in this study. Will these results hold for those locations? With respect to the stability of the most sensitive zone in the receptive field, existing data suggest that the majority also have a set of stable inputs, but simply lack the responsiveness to be included. For example, our 13 locations with sensitivities judged as weaker than clearly cutaneous also largely maintained a constant region in their most sensitive zones, and this finding is all the more surprising because of the difficulty in precisely localizing a receptive field in a location of this sensitivity. A prior study (Blake et al. 2005) found constancy in a portion of the receptive fields as well, although the time course was much shorter. However, not all locations have stable inputs. Especially in the case of focal hand dystonia, a substitution of inputs has been observed (Blake et al. 2002a).

The finding of spatial invariance of a portion of the most sensitive inputs suggests that the map of the most sensitive inputs at each cortical location is not plastic during normal learning‐induced plasticity. The receptive field size and sensitivity can be scaled up, and down, as the animal forms new associations between sensory stimuli and reward or omission of reward (Spingath et al. 2011). A number of published map plasticity studies apparently contradict this finding (Jenkins et al. 1990; Recanzone et al. 1992a; Wang et al. 1995a; Xerri et al. 1996). Much of this apparent contradiction can be explained by penetrations mapped as “deep” actually containing cutaneous inputs with low sensitivity, and of penetrations that have most sensitive inputs that are proprioceptive that gain lower sensitivity cutaneous inputs. The classification of cortical neurons as deep or cutaneous was used in the earliest studies of somatosensory cortex (Mountcastle 1957; Powell and Mountcastle 1959). Stimuli were used that moved the skin, but arguably did not sufficiently deform the viscera to cause a response in any neurons but low‐threshold mechanoreceptors. Later studies use the criteria of responding to “just visible” indentations of the skin as determining whether inputs are cutaneous or deep. One of us (D.B.) built a small, hand‐held, piezoelectric skin tapper that delivered taps up to 40 *μ*m in magnitude, and compared the audible responses to the tapper to the judgments of responses to “just visible” skin indentations. The problem, really, is not in making those judgments, but in cortical gain. Every peripheral cutaneous neuron (SA1, RA, and PC) will respond to 40 *μ*m skin taps consistently. However, neurons in cortex may respond with greatly reduced gain, so that even 500 *μ*m skin taps only elicit inconsistent responses. The cortical neuron can receive input from cutaneous sources, but respond so weakly it is judged to be a deep response. Also, it is difficult to assess proprioceptive responses in a mapping study in an anesthetized animal, as the muscle tone is inadequate to excite muscle spindles. Only because we created a recording method that enabled us to sample from the same cortical locations over time, in the awake animal, allows the distinction between these classes of map plasticity and neural sensitivity to be formed. The population response, however, will be altered entirely according to the findings of those map plasticity studies. The results are not wrong. However, it is changes in sensitivity, and changes in receptive field size, that account for those changes, and not changes in the map defined by the most sensitive inputs at each cortical location during learning‐induced plasticity.

These ideas challenge and refine ideas on learning‐induced plasticity in sensory cortex. Prior to the studies on learning‐induced plasticity, the field was dominated by the ideology that cortical neurons establish their connectivity during the critical period in development, and thereafter lack sensitivity to sensory perturbations that would have caused plasticity during development (Hubel and Wiesel 1970). These ideas, however, were challenged in studies of denervation (Merzenich et al. 1984), which leads to plasticity in cortex as well as in the dorsal horn (Basbaum and Wall 1976). Further challenge came from studies of digital syndactyly (Allard et al. 1991), which showed that with experience‐dependent alteration of sensory inputs in the adult, the topography of the cortical map was altered. Next, studies using operant training showed that the area representing behavioral relevant epithelial inputs would undergo experience‐dependent expansion (Jenkins et al. 1990; Recanzone et al. 1992b; Wang et al. 1995b; Xerri et al. 1999). Work in visual and somatosensory cortex corroborated a cortical basis for this phenomenology (Darian‐Smith and Gilbert 1995; Wang et al. 1995b; Blake et al. 2002a). In parallel, other work on learning‐induced plasticity followed a Pavlovian methodology of seeking a basis for plasticity in association with reinforcement (Weinberger 1995, 2004). Our work builds upon and extends this body of work by establishing that association with reinforcement leads to a nonselective response enhancement in sensory cortex (Blake et al. 2002b, 2005, 2006; Carpenter‐Hyland et al. 2010; Spingath et al. 2011, 2013). In addition, associating a sensory stimulus with omission of reward leads to a selective response suppression. The current work establishes that locations in sensory cortex maintain their most sensitive inputs throughout learning, which implies the gross topography of cortical maps does not change from learning‐induced plasticity. Similar observations have been made using coarser technology in vision (Cheng et al. 2001; De Beeck et al. 2008). The learning observed in the current study occurs through changes in the weaker inputs in cortical receptive fields, and by changes in the response strength and receptive field size driven by association with reward or omission of reward. Work on learning‐induced plasticity in primary visual cortex can be interpreted as caused by the same effects (Schoups et al. 2001; Ghose et al. 2002). Accordingly, we understand learning‐induced sensory cortex plasticity in terms of not shifting maps, but of changes in responsiveness and receptive field size in maps receiving their most sensitive inputs from the same locations in the sensory epithelia.

The stability of the most sensitive inputs plays a role in the transformation of sensory stimuli from input to central representations. Such stability can, under certain conditions, eradicate the need to consider reconstruction errors in learning and provide a substrate for perceptual constancy.

## Conflict of Interest

None declared.

## References

[phy212623-bib-0001] Allard, T. , S. A. Clark , W. M. Jenkins , and M. M. Merzenich . 1991 Reorganization of somatosensory area 3b representations in adult owl monkeys after digital syndactyly. J. Neurophysiol. 66:1048–1058. 1753275 10.1152/jn.1991.66.3.1048

[phy212623-bib-0002] Basbaum, A. I. , and P. D. Wall . 1976 Chronic changes in the response of cells in adult cat dorsal horn following partial deafferentation: the appearance of responding cells in a previously non‐responsive region. Brain Res. 116:181–204. 974771 10.1016/0006-8993(76)90899-4

[phy212623-bib-0003] Blake, D. T. , N. N. Byl , S. Cheung , P. Bedenbaugh , S. Nagarajan , M. Lamb , et al. 2002a Sensory representation abnormalities that parallel focal hand dystonia in a primate model. Somatosens. Mot. Res. 19:347–357. 12590836 10.1080/0899022021000037827 PMC2826977

[phy212623-bib-0004] Blake, D. T. , F. Strata , A. K. Churchland , and M. M. Merzenich . 2002b Neural correlates of instrumental learning in primary auditory cortex. Proc. Nat. Acad. Sci. U. S. A. 99:10114–10119. 10.1073/pnas.092278099 PMC126633 12119383

[phy212623-bib-0005] Blake, D. , F. Strata , R. Kempter , and M. Merzenich . 2005 Experience‐dependent plasticity in S1 caused by noncoincident inputs. J. Neurophysiol. 94:2239–2250. 16105958 10.1152/jn.00172.2005 PMC2826984

[phy212623-bib-0006] Blake, D. , M. Heiser , M. Caywood , and M. Merzenich . 2006 Experience‐dependent adult cortical plasticity requires cognitive association between sensation and reward. Neuron 52:371–381. 17046698 10.1016/j.neuron.2006.08.009 PMC2826987

[phy212623-bib-0007] Boulton, A. A. , G. B. Baker , and C. H. Vanderwolf . 1990 Neurophysiological techniques: applications to neural systems Humana Press, Clifton, NJ.

[phy212623-bib-0008] Buonomano, D. V. , and M. M. Merzenich . 1998 Cortical plasticity: from synapses to maps. Annu. Rev. Neurosci. 21:97–102. 9530495 10.1146/annurev.neuro.21.1.149

[phy212623-bib-0009] Carpenter‐Hyland, E. , T. Plummer , A. Vazdarjanova , and D. Blake . 2010 Arc expression and neuroplasticity in primary auditory cortex during initial learning are inversely related to neural activity. Proc. Nat. Acad. Sci. U. S. A. 107:14828–14832. 10.1073/pnas.1008604107 PMC2930446 20675582

[phy212623-bib-0010] de Charms, R. C. , D. T. Blake , and M. M. Merzenich . 1999 A multielectrode implant device for the cerebral cortex. J. Neurosci. Methods 93:27–35. 10598862 10.1016/s0165-0270(99)00087-4

[phy212623-bib-0011] Cheng, K. , R. Waggoner , and K. Tanaka . 2001 Human ocular dominance columns as revealed by high‐field functional magnetic resonance imaging. Neuron 32:359–374. 11684004 10.1016/s0896-6273(01)00477-9

[phy212623-bib-0012] Chubbuck, J. 1966 Small motion biological stimulator. Johns Hopkins APL Tech 5:18–23.

[phy212623-bib-0013] Collins, C. , D. Airey , N. Young , D. Leitch , and J. Kaas . 2010 Neuron densities vary across and within cortical areas in primates. Proc. Nat. Acad. Sci. U. S. A. 107:15927–15932. 10.1073/pnas.1010356107 PMC2936588 20798050

[phy212623-bib-0014] Darian‐Smith, C. , and C. D. Gilbert . 1995 Topographic reorganization in the striate cortex of the adult cat and monkey is cortically mediated. J. Neurosci. 15:1631–1647. 7891124 10.1523/JNEUROSCI.15-03-01631.1995 PMC6578152

[phy212623-bib-0015] De Beeck, H. P. O. , J. A. Deutsch , W. Vanduffel , N. G. Kanwisher , and J. J. DiCarlo . 2008 A stable topography of selectivity for unfamiliar shape classes in monkey inferior temporal cortex. Cereb. Cortex 18:1676–1694. 18033769 10.1093/cercor/bhm196 PMC2731473

[phy212623-bib-0016] Ghose, G. , T. Yang , and J. Maunsell . 2002 Physiological correlates of perceptual learning in monkey V1 and V2. J. Neurophysiol. 87:1867–1888. 11929908 10.1152/jn.00690.2001

[phy212623-bib-0017] Hubel, D. H. 1957 Tungsten microelectrode for recording from single units. Science 125:549–550. 17793797 10.1126/science.125.3247.549

[phy212623-bib-0018] Hubel, D. , and T. Wiesel . 1970 The period of susceptibility to the physiological effects of unilateral eye closure in kittens. J. Physiol. 206:419–436. 5498493 10.1113/jphysiol.1970.sp009022 PMC1348655

[phy212623-bib-0019] Jenkins, W. M. , M. M. Merzenich , M. T. Ochs , T. Allard , and E. Guic‐Robles . 1990 Functional reorganization of primary somatosensory cortex in adult owl monkeys after behaviorally controlled tactile stimulation. J. Neurophysiol. 63:82–104. 2299388 10.1152/jn.1990.63.1.82

[phy212623-bib-0020] Johansson, R. 1978 Tactile sensibility in the human hand: receptive field characteristics of mechanoreceptive units in the glabrous skin area. J. Physiol. 281:101–125. 702358 10.1113/jphysiol.1978.sp012411 PMC1282686

[phy212623-bib-0021] Killebrew, J. , S. Bensmaia , J. Dammann , P. Denchev , S. Hsiao , J. Craig , et al. 2007 A dense array stimulator to generate arbitrary spatio‐temporal tactile stimuli. J. Neurosci. Methods 161:62–74. 17134760 10.1016/j.jneumeth.2006.10.012 PMC1851669

[phy212623-bib-0022] Merzenich, M. M. , J. H. Kaas , M. Sur , and C. S. Lin . 1978 Double representation of the body surface within cytoarchitectonic areas 3b and 1 in si in the owl monkey (*Aotus trivirgatus*). J. Comp. Neurol. 181:41–73. 98537 10.1002/cne.901810104

[phy212623-bib-0023] Merzenich, M. , R. Nelson , M. Stryker , M. Cynader , A. Schoppmann , and J. Zook . 1984 Somatosensory cortical map changes following digit amputation in adult monkeys. J. Comp. Neurol. 224:591–605. 6725633 10.1002/cne.902240408

[phy212623-bib-0024] Miyakawa, N. , N. Katsumata , D. Blake , M. Merzenich , and M. Tanifuji . 2012 High‐density multielectrode array with independently maneuverable electrodes and silicone oil fluid isolation system for chronic recording from macaque monkey. J. Neurosci. Methods 211:114–124. 22939944 10.1016/j.jneumeth.2012.08.019

[phy212623-bib-0025] Mountcastle, V. B. 1957 Modality and topographic properties of single neurons of the cat's somatic sensory cortex. J. Neurophysiol. 20:408–434. 13439410 10.1152/jn.1957.20.4.408

[phy212623-bib-0026] Pei, Y. , P. Denchev , S. Hsiao , J. Craig , and S. Bensmaia . 2009 Convergence of submodality‐specific input onto neurons in primary somatosensory cortex. J. Neurophysiol. 102:1843–1853. 19535484 10.1152/jn.00235.2009 PMC2746774

[phy212623-bib-0027] Powell, T. P. , and V. B. Mountcastle . 1959 Some aspects of the functional organization of the cortex of the postcentral gyrus of the monkey: a correlation of findings obtained in a single unit analysis with cytoarchitecture. Bull. Johns Hopkins Hosp. 105:133–162. 14434571

[phy212623-bib-0028] Recanzone, G. H. , W. M. Jenkins , G. T. Hradek , and M. M. Merzenich . 1992a Progressive improvement in discriminative abilities in adult owl monkeys performing a tactile frequency discrimination task. J. Neurophysiol. 67:1015–1030. 1597695 10.1152/jn.1992.67.5.1015

[phy212623-bib-0029] Recanzone, G. H. , M. M. Merzenich , W. M. Jenkins , K. A. Grajski , and H. R. Dinse . 1992b Topographic reorganization of the hand representation in cortical area 3b owl monkeys trained in a frequency‐discrimination task. J. Neurophysiol. 67:1031–1056. 1597696 10.1152/jn.1992.67.5.1031

[phy212623-bib-0030] Schmidt, E. M. , J. S. McIntosh , and M. J. Bak . 1988 Long‐term implants of parylene‐c coated microelectrodes. Med. Biol. Eng. Comput. 26:96–101. 3199908 10.1007/BF02441836

[phy212623-bib-0031] Schoups, A. , R. Vogels , N. Qian , and G. Orban . 2001 Practising orientation identification improves orientation coding in V1 neurons. Nature 412:549–553. 11484056 10.1038/35087601

[phy212623-bib-0032] Spingath, E. , H. Kang , T. Plummer , and D. Blake . 2011 Different neuroplasticity for task targets and distractors. PLoS ONE 6:e15342. 21297962 10.1371/journal.pone.0015342 PMC3031528

[phy212623-bib-0033] Spingath, E. , H. Kang , and D. Blake . 2013 Task‐dependent modulation of SI physiological responses to targets and distractors. J. Neurophysiol. 109:1036–1044. 23197458 10.1152/jn.00385.2012 PMC3569122

[phy212623-bib-0034] Wang, X. , M. M. Merzenich , R. Beitel , and C. E. Schreiner . 1995a Representation of a species‐specific vocalization in the primary auditory cortex of the common marmoset: temporal and spectral characteristics. J. Neurophysiol. 74:2685–2706. 8747224 10.1152/jn.1995.74.6.2685

[phy212623-bib-0035] Wang, X. , M. M. Merzenich , K. Sameshima , and W. M. Jenkins . 1995b Remodelling of hand representation in adult cortex determined by timing of tactile stimulation. Nature 378:71–75. 7477291 10.1038/378071a0

[phy212623-bib-0036] Weinberger, N. 1995 Dynamic regulation of receptive fields and maps in the adult sensory cortex. Annu. Rev. Neurosci. 18:129–158. 7605058 10.1146/annurev.ne.18.030195.001021 PMC3621971

[phy212623-bib-0037] Weinberger, N. 2004 Specific long‐term memory traces in primary auditory cortex. Nat. Rev. Neurosci. 5:279–290. 15034553 10.1038/nrn1366 PMC3590000

[phy212623-bib-0038] Xerri, C. , J. O. Coq , M. M. Merzenich , and W. M. Jenkins . 1996 Experience‐induced plasticity of cutaneous maps in the primary somatosensory cortex of adult monkeys and rats. J. Physiol. Paris 90:277–287. 9116682 10.1016/s0928-4257(97)81438-6

[phy212623-bib-0039] Xerri, C. , M. M. Merzenich , W. Jenkins , and S. Santucci . 1999 Representational plasticity in cortical area 3b paralleling tactual‐motor skill acquisition in adult monkeys. Cereb. Cortex 9:264–276. 10355907 10.1093/cercor/9.3.264

[phy212623-bib-0040] Xerri, C. , S. Bourgeon , and J. Coq . 2005 Perceptual context‐dependent remodeling of the forepaw map in the SI cortex of rats trained on tactile discrimination. Behav. Brain Res. 162:207–221. 15923046 10.1016/j.bbr.2005.03.003

